# Blood pressure and cholesterol measurements in primary care: cross-sectional analyses in a dynamic cohort

**DOI:** 10.3399/BJGPO.2021.0131

**Published:** 2022-03-23

**Authors:** Annemarijn R de Boer, Monika Hollander, Ineke van Dis, Frank LJ Visseren, Michiel L Bots, Ilonca Vaartjes

**Affiliations:** 1 Julius Center for Health Sciences and Primary Care, University Medical Center Utrecht, Utrecht University, Utrecht, The Netherlands; 2 Dutch Heart Foundation, The Hague, The Netherlands; 3 Department of Vascular Medicine, University Medical Center Utrecht, Utrecht University, Utrecht, The Netherlands

**Keywords:** blood pressure, cardiovascular diseases, cholesterol, CVRM guideline, diabetes mellitus, electronic health records, general practice, heart disease risk factors, Netherlands, trends

## Abstract

**Background:**

Guidelines on cardiovascular risk management (CVRM) recommend blood pressure (BP) and cholesterol measurements every 5 years in men aged ≥40 years and (post-menopausal) women aged ≥50 years.

**Aim:**

To evaluate CVRM guideline implementation.

**Design & setting:**

Cross-sectional analyses in a dynamic cohort using primary care electronic health record (EHR) data from the Julius General Practitioners’ Network (JGPN) (*n* = 388 929).

**Method:**

Trends (2008–2018) were assessed in the proportion of patients with at least one measurement (BP and cholesterol) every 1, 2, and 5 years, in those with:

1. a history of cardiovascular disease (CVD) and diabetes mellitus (DM);

2. a history of DM only;

3. a history of CVD only;

4. a cardiovascular risk assessment (CRA) indication based on other medical history, or;

5. no CRA indication.

Trends were evaluated over time using logistic regression mixed-model analyses.

**Results:**

Trends in annual BP and cholesterol measurement increased for patients with a history of CVD from 37.0% to 48.4% (*P*<0.001) and 25.8% to 40.2% (*P*<0.001). In the 5-year window from 2014–2018, BP and cholesterol measurements were performed respectively in 78.5% and 74.1% of all men aged ≥40 years and 82.2% and 78.5% of all women aged ≥50 years. Least measured were patients without a CRA indication (men 60.2% and 62.4%; women 55.5% and 59.3%).

**Conclusion:**

The fairly high frequency of CVRM measurements available in the EHR of patients in primary care suggests an adequate implementation of the CVRM guideline. As nearly all individuals visit the GP at least once within a 5-year time window, improvement of CVRM remains possible, especially in those without a CRA indication.

## How this fits in

Guidelines on CVRM recommend BP and cholesterol measurements every 5 years in men aged ≥40 years and (post-menopausal) women aged ≥50 years. This study shows that a majority of these men and women (74–82%) receive these measurements in primary care. Least often measured were patients without an indication for CRA other than age (55–62%). As nearly all individuals visit the GP at least once within a 5-year time window, improvement of CVRM remains very well possible, especially in those without a CRA indication.

## Introduction

CVD is the leading cause of disability and death in the world.^1–3,3^ A large proportion of CVD can potentially be prevented by controlling modifiable risk factors such as elevated BP, raised cholesterol, smoking, or obesity.^4^

Guidelines on CVRM were introduced to improve detection and treatment of patients with already established CVD and those at high risk of CVD.^5–8,7,8^ Decisions about initiating treatment are made on the basis of — among other things — an individual patient’s cardiovascular risk, often expressed as 10-year risk of developing non-fatal or fatal CVD. To monitor cardiovascular risk, guidelines advice to perform regular CRA, which includes the measurement of two important CVD risk factors: BP and cholesterol.^5–8,7,8^

Evidence regarding the optimal frequency of BP and cholesterol measurements for CVRM is still inconclusive, illustrated by the variation in wording in the various guidelines regarding this topic, sometimes only expressed as ‘regularly’.^5–9,7,8,9^ The most recent revision of the Dutch CVRM guideline in 2019, endorsed by both GPs and other medical specialists, suggests the performance of a CRA every 5 years in all men aged ≥40 years and all post-menopausal women or women aged ≥50 years.^10^ Until 2019, although the guideline specified the population in which a CRA should be performed, the frequency of this assessment was not defined.^11–13,13^

Given the change in recommendation, this study aimed to evaluate the implementation of the CVRM guideline up to 2018, and recommend in which patients guideline adherence should be improved. The study proposes to do this by assessing temporal trends in BP and cholesterol measurements in primary care using routine EHR data in patients with and without an indication for CRA.

## Method

### Data source and study design

Data were obtained from the JGPN database. The database contains routine clinical care data anonymously extracted from structured fields within the EHRs from all patients registered in 72 general practices in the city of Utrecht and its vicinity in The Netherlands.^14^ The JGPN can be seen as a dynamic cohort, since the membership of the cohort is not fixed. Patients enter the JGPN cohort by being born in or moving to the catchment area of one of the JGPN general practices, and leave the cohort by dying or moving away. In The Netherlands, all inhabitants (except older people residing in nursing homes) are obliged to register at a general practice and have access to health care, since healthcare insurance is mandatory. GPs act as gatekeepers to hospital care and play a key role in CVRM. The JGPN population is considered representative of the Dutch population with regard to sex and age.^14^

### Study population

All patients aged ≥18 years, registered at one of the affiliated general practices of the JGPN between 1 January 2008 and 31 December 2018, and who contributed at least 1 calendar year to the database were included.

### Outcome measures

For both BP and cholesterol, all patients were defined as ‘measured’ or ‘not measured’ for each study year. A patient was defined as ‘measured’ for BP if that patient had at least one registered systolic or diastolic BP measurement during that study year. A patient was defined as ‘measured’ for cholesterol if that patient had at least one registered measurement of either low-density lipoprotein (LDL), high-density lipoprotein (HDL), total cholesterol, triglycerides, or total cholesterol/HDL ratio during that study year. If patients had multiple registered measurements in a year, the first measurement was included in the analyses.

### Determinants

Information about sex, age, medical history, and antihypertensive or lipid-lowering medication use was extracted from the EHR for each patient. Medical history was defined based on the International Classification of Primary Care codes (see Supplementary Table S1). Medication use was classified according to Anatomical Therapeutic Chemical Classification System.

Each patient was classified into one of five categories based on their medical history:

1. a history of CVD and DM;

2. a history of DM without CVD;

3. a history of CVD without DM;

4. a CRA indicated on the basis of other medical history; or

5. no CRA indicated.

Since patients with DM most commonly receive 3-monthly check-ups within primary care as part of a disease management programme in The Netherlands including BP measurement, they were analysed as a separate group. Patients were classified as having a history of CVD based on the definition of CVD mentioned in the CVRM guideline used in primary care in The Netherlands between 2012 and 2019: myocardial infarction, angina pectoris, heart failure, stroke/cerebrovascular accident, transient ischaemic attack, peripheral arterial disease, aortic aneurysm.^12^ The ‘CRA indicated’ category was defined on the basis of the 2012 CVRM guideline and comprised patients with certain medical history and characteristics on the basis of which they were suspected to be at high cardiovascular risk, and for which the guideline recommends regular CRA: rheumatoid arthritis, pre-eclampsia, pregnancy-induced DM, family history of CVD, obesity, smoking, hypertension, dyslipidaemia, chronic kidney disease, and use of antihypertensive or lipid-lowering medication (see Supplementary Table S2 for details). If patients could not be classified into categories 1–4 they were categorised as ‘no CRA indicated’.

### Statistical analyses

First, the characteristics of the study population were described for every other year.

Second, to analyse trends over time, the proportion of men and women with a registered measurement was calculated (separately for each of BP and cholesterol ) for each year of the study period. The five categories of medical history, sex, and age categories (18–39, 40–49, 50–59, 60–69, and ≥70 years) were stratified for. Next, the proportion of men and women with a registered measurement at least once in 2 years and once in 5 years was calculated. Only patients who contributed a consecutive 2 and consecutive 5 calendar years to the database were included. To investigate whether trends over time were statistically significant, logistic regression mixed-model analyses were performed with random effects for time (in years) and general practice to account for clustering. To adjust for possible confounding, age and sex were added to the model. Time and age were standardised and the optimiser ‘Neal-Head’ from the R package ‘lme4’ was used to allow for model convergence.^15^ To investigate differences in temporal trends between the five categories of medical history, an interaction term was added between time and category of medical history to the model (reference group: no CRA indicated). If significant, a stratified analysis was performed for each category of medical history to investigate whether trends over time in these subgroups were statistically significant. The likelihood ratio test was used to compute *P* values. A two-sided *P* value <0.05 was deemed statistically significant.

Third, to evaluate the new recommendation of the 2019 CVRM guideline, the proportion of all men aged ≥40 years and women aged ≥50 years with at least one measurement between 2014 and 2018 was calculated, and category of medical history was stratified for.

All analyses and visualisations were performed in R (version 4.3).

### Privacy and ethics procedures

Research with data from the JGPN is observational and non-interventional; the data used contain non-identifiable information. Therefore, according to Dutch laws on privacy and research on human subjects (WMO = Wet medisch-wetenschappelijk onderzoek met mensen *[Medical Research Involving Human Subjects Act])*, the Medical Ethics Committees in The Netherlands do not consider this research as subject to the WMO conditions. Researchers need to conform to privacy legislation.^14^

## Results

Between 1 January 2008 and 31 December 2018, 388 929 patients aged ≥18 years were registered within the JGPN database with a total of 2 271 084 observed patient years (median follow-up time = 5 years, interquartile range [IQR] = 2–9 years). Table 1 presents characteristics of the studied population.

**Table 1:** Baseline characteristics of studied population

	2008*n* = 158 916	2010*n* = 169 178	2012*n* = 188 073	2014*n* = 244 390	2016*n* = 261 283	2018*n* = 190 528
Men, *n* (%)	75 694 (47.6)	80 777 (47.7)	89 986 (47.8)	117 063 (47.9)	125 514 (48.0)	92 078 (48.3)
Mean age, years (SD)	45.5 (17.1)	45.7 (17.2)	46.1 (17.3)	46.4 (17.6)	46.2 (17.6)	46.2 (17.8)
Previous CVD, *n* (%)	9394 (5.9)	11 255 (6.7)	13 226 (7.0)	16 965 (6.9)	18 974 (7.3)	13 852 (7.3)
DM, *n* (%)	8976 (5.6)	10 640 (6.3)	12 345 (6.6)	15 766 (6.5)	17 478 (6.7)	12 786 (6.7)
CVD and DM, *n* (%)	2205 (1.4)	2846 (1.7)	3460 (1.8)	4553 (1.9)	5188 (2.0)	3732 (2.0)
CRA indication,^a^ *n* (%)	25 355 (16.0)	32 054 (18.9)	39 882 (21.2)	53 391 (21.8)	61 000 (23.3)	45 759 (24.0)

^a^Patients were classified as ‘CRA indication’ if they had no history of CVD or DM, but did have a history of one of the following: rheumatoid arthritis, pre-eclampsia, pregnancy-induced DM, family history of CVD, obesity, smoking, hypertension, dyslipidaemia, chronic kidney disease, use of antihypertensive or lipid-lowering medication.

DM = diabetes mellitus. CRA = cardiovascular risk assessment. CVD = cardiovascular disease. SD = standard deviation.

### Trends in measurements

For both BP and cholesterol measurements, the proportion of men and women (aged ≥18 years) with a registered measurement increased between 2008 and 2018: from 12.4% to 17.8% for BP, and 11.1% to 15.4% for cholesterol in men; and 15.5% to 20.6% for BP, and from 12.1% to 16.1% for cholesterol in women (see Supplementary Figure S1 for details). Consistently, a registered measurement was more common in women than in men, and a registered BP measurement was more commonly performed than a cholesterol measurement (Supplementary Figure S1).

Older individuals, irrespective of medical history, more often had a measurement compared with younger individuals. Furthermore, the largest increase in proportion of patients with an annual registered measurement over time was seen in older individuals (Supplementary Figure S2).

**Figure 1:**
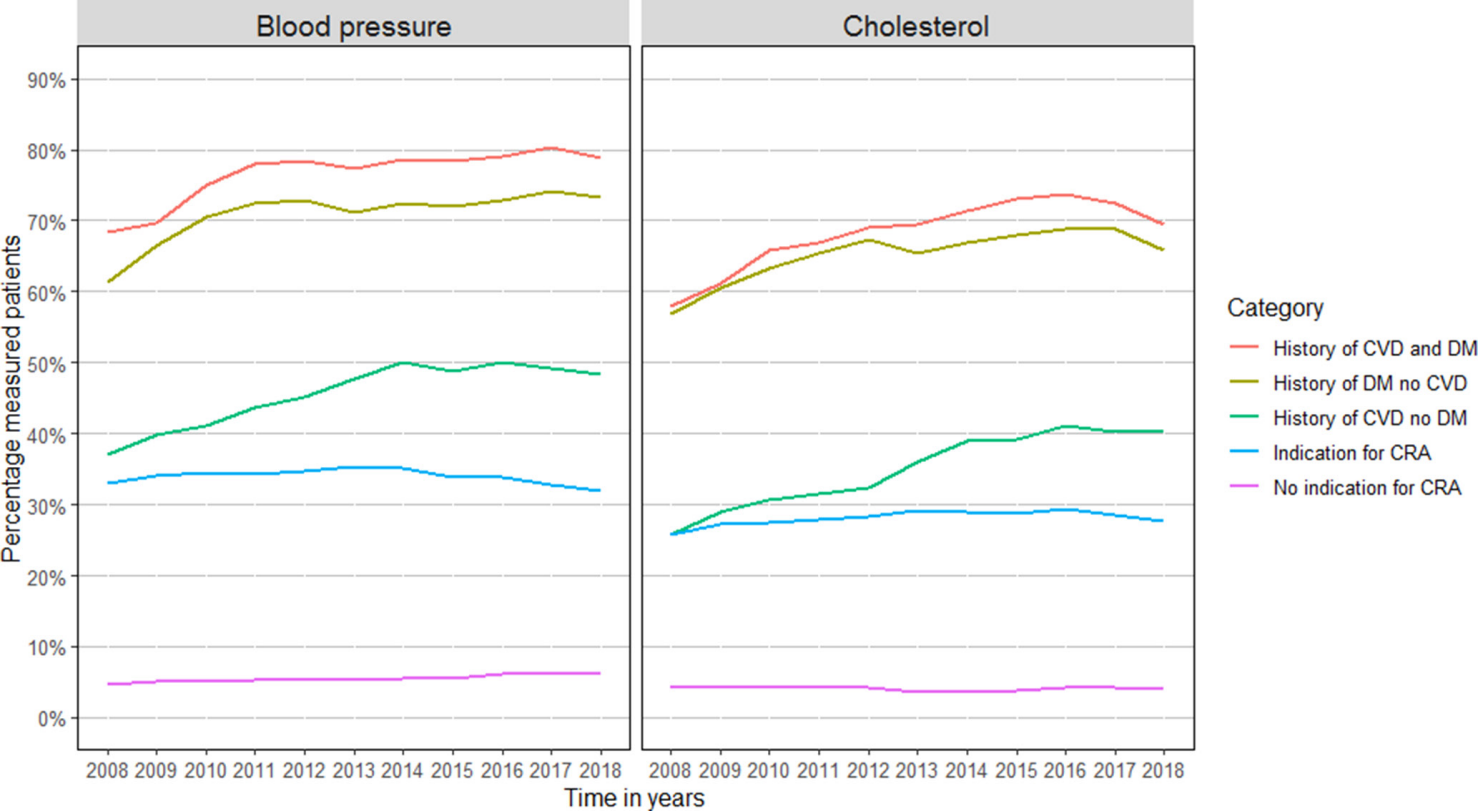
Trends in annual BP and cholesterol measurement stratified for medical history. Patients were classified as ‘Indication for CRA’ if they did not have a history of CVD or DM, but did have a history of at least one of the following: rheumatoid arthritis, pre-eclampsia, pregnancy-induced DM, family history of CVD, obesity, smoking, hypertension, dyslipidaemia, chronic kidney disease, use of antihypertensive or lipid-lowering medication. CRA = cardiovascular risk assessment. CVD = cardiovascular disease. DM = diabetes mellitus.

Across all disease groups, there is a clear age effect: the proportion of annual measurements is much lower in younger age groups, even where there is a clear indication such as DM, CVD, or both (Figures 1 2).

**Figure 2:**
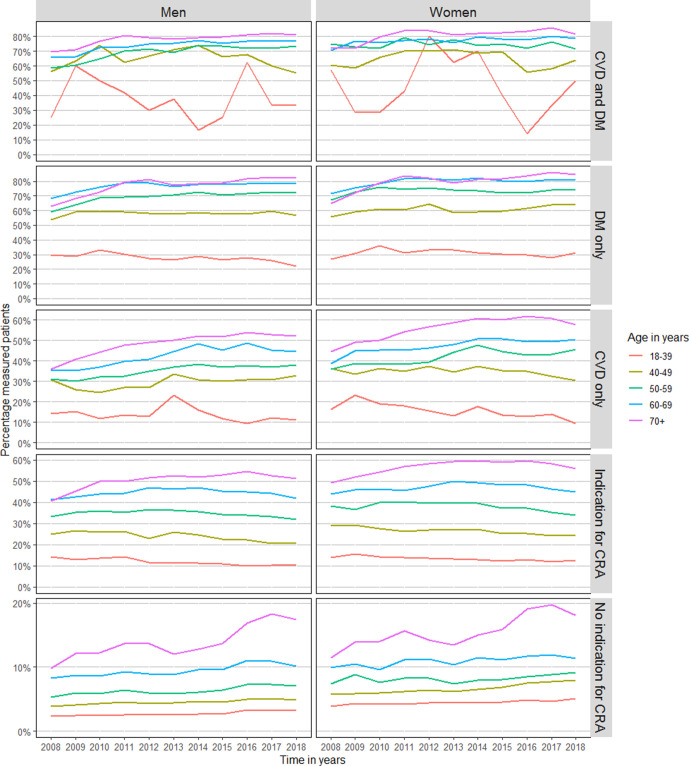
Sex and age stratified trends in annual BP measurement in patients with a history of CVD, DM, indication for CRA, or without an indication for CRA. Patients were classified as ‘Indication for CRA’ if they did not have a history of CVD or DM, but did have a history of one of the following: rheumatoid arthritis, pre-eclampsia, pregnancy-induced DM, family history of CVD, obesity, smoking, hypertension, dyslipidaemia, chronic kidney disease, use of antihypertensive or lipid-lowering medication. CRA = cardiovascular risk assessment. CVD = cardiovascular disease. DM = diabetes mellitus.

**Figure 3:**
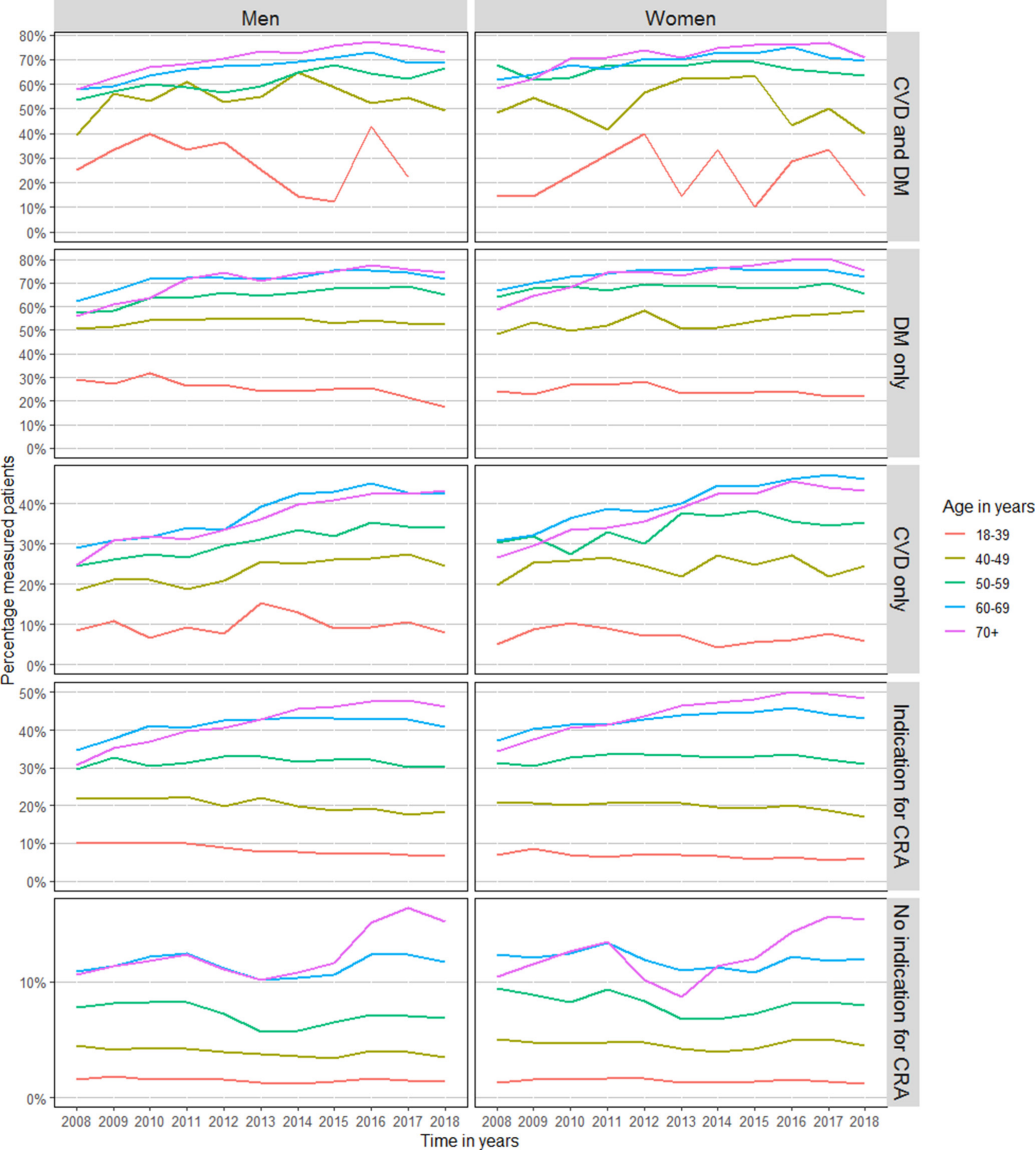
Sex and age stratified trends in annual cholesterol measurement in patients with a history of CVD, DM, indication for CRA, or without an indication for CRA. CRA = cardiovascular risk assessment. Patients were classified as ‘Indication for CRA’ if they did not have a history of CVD or DM, but did have a history of one of the following: rheumatoid arthritis, pre-eclampsia, pregnancy-induced DM, family history of CVD, obesity, smoking, hypertension, dyslipidaemia, chronic kidney disease, use of antihypertensive or lipid-lowering medication. CVD = cardiovascular disease. DM = diabetes mellitus.

Results based on subgroups are presented in Table 2. In patients with a clear disease entity (such as CVD, or DM) the proportion of patients with an annual measurement and measurements every 2 years significantly increased (Figure 3, Supplementary Figure S3). A significant increase for measurements every 5 years was seen in patients with CVD, whereas in patients with an indication for CRA the trend decreased (Supplementary Figure S4).

**Table 2:** Trends in BP and cholesterol measurements between 2008 and 2018

Annual measurements (2008–2018)		
	BP	Cholesterol
CVD and DM (%)	68.5–78.7 (*P*<0.001)	57.9–69.6 (*P*<0.001)
DM only (%)	61.5–73.3 (*P* = 0.002)	56.8–65.8 (*P* = 0.001)
CVD only (%)	37.0–48.4 (*P*<0.001)	25.8–40.2 (*P*<0.001)
Indication for CRA (%)	33.0–31.9 (*P* = 0.37)	25.7–27.7 (*P* = 0.43)
No indication for CRA (%)	4.7–6.2 (*P* = 0.034)	4.3–3.9 (*P* = 0.53)
**Measurements every 2 years (2009–2010 and 2017–2018)**		
	BP	Cholesterol
CVD and DM (%)	80.8–87.6 (*P*<0.001)	75.9–82.7 (*P*<0.001)
DM only (%)	76.2–82.2 (*P* = 0.006)	73.2–79.3 (*P*<0.001)
CVD only (%)	54.5–64.2 (*P*<0.001)	45.7–56.8 (*P*<0.001)
Indication for CRA (%)	47.0–45.8 (*P* = 0.99)	41.7–41.1 (*P* = 0.85)
No indication for CRA (%)	9.2–11.1 (*P* = 0.36)	8.0–7.4 (*P* = 0.12)
**Measurements every 5 years (2009–2013 and 2014–2018)**		
	BP	Cholesterol
CVD and DM (%)	93.2–93.7 (*P* = 0.073)	88.1–89.1 (*P* = 0.082)
DM only (%)	88.5–88.9 (*P* = 0.65)	84.8–85.8 (*P* = 0.057)
CVD only (%)	78.6–81.2 (*P*<0.001)	70.6–74.2 (*P* = 0.002)
Indication for CRA (%)	68.5–66.2 (*P* = 0.023)	62.7–60.5 (*P* = 0.009)
No indication for CRA (%)	19.6–22.4 (*P* = 0.10)	16.3–16.0 (*P* = 0.12)

Patients were classified as ‘Indication for CRA’ if they did not have a history of CVD or DM, but did have a history of one of the following: rheumatoid arthritis, pre-eclampsia, pregnancy-induced DM, family history of CVD, obesity, smoking, hypertension, dyslipidaemia, chronic kidney disease, use of antihypertensive or lipid-lowering medication.

BP = blood pressure. CRA = cardiovascular risk assessment. CVD = cardiovascular disease.

### Evaluation of the 2019 CVRM guideline

**Figure 4:**
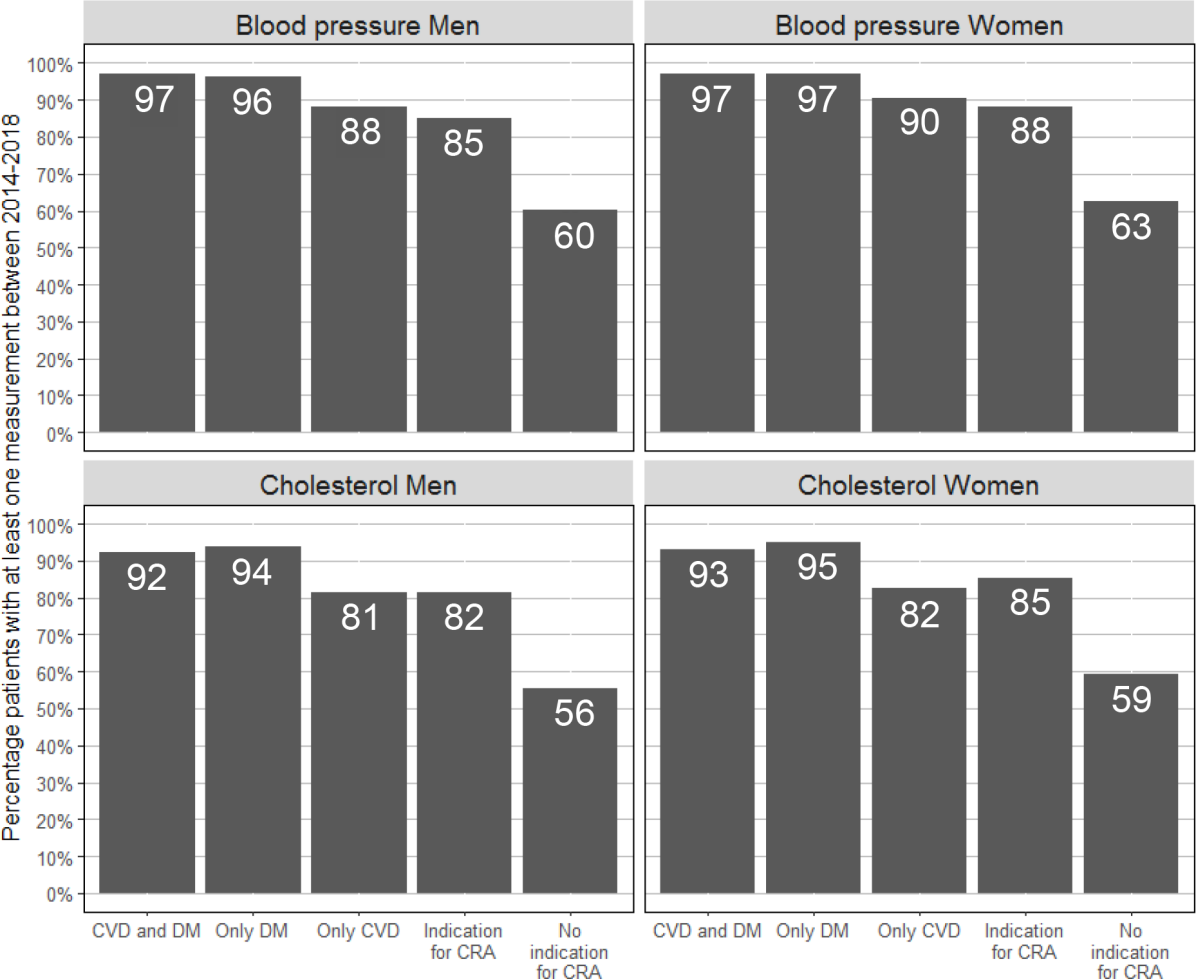
Men aged ≥40 years and women aged ≥50 years with at least one measurement between 2014 and 2018. CRA = cardiovascular risk assessment. Patients were classified as ‘Indication for CRA’ if they did not have a history of CVD or DM, but did have a history of one of the following: rheumatoid arthritis, pre-eclampsia, pregnancy-induced DM, family history of CVD, obesity, smoking, hypertension, dyslipidaemia, chronic kidney disease, use of antihypertensive or lipid-lowering medication. CVD = cardiovascular disease. DM = diabetes mellitus.

In the 5 years between 2014 and 2018, 78.5% of men aged ≥40 years received at least one BP measurement and 74.1% received at least one cholesterol measurement. For women, this was 82.2% for BP and 78.5% for cholesterol. Figure 4 shows the percentage measured patients stratified for category of medical history. The percentage of measured patients was highest in patients with DM, and lowest in those without an indication for CRA.

## Discussion

### Summary

Overall, the proportion of individuals in primary care with a BP and a cholesterol measurement increased between 2008 and 2018.

This occurred in men and women, especially in those aged ≥50 years. Furthermore, it increased in those groups of patients in which the prevailing guidelines recommended taking such measurements. In the subgroup with a CRA indication but without a clear disease entity (CVD, DM), no change was observed between 2008 and 2018. Importantly, over 80% of men aged ≥40 years and women aged ≥50 years with a CRA indication in general practice had at least one BP or cholesterol measurement taken in a 5-year period. Moreover, around 50–60% of those without any indication had these measurements done.

### Strengths and limitations

The strengths of the present study are the large dynamic cohort with comprehensive data representative of routine clinical care. Some aspects need consideration before the interpretation of the results. A limitation that is inherent to using EHR data is that the quality of the data depends on the accuracy of recording on the part of the GP. Misclassification of patients into one of the five categories of medical history could have occurred if medical history was not properly recorded. This would most likely work in a direction that absence of a disease is not registered, and thus individuals are assigned to the ‘no indication’ category. Please note that for this study, only data registered within fixed fields of the EHRs, but not those in the free-text fields, was used. Since physicians sometimes write BP measurement results in free-text fields, this could have led to an underestimation of the proportion of patients with a BP measurement. Cholesterol is only registered in fixed field data. Since trends in BP and cholesterol measurements were very similar, it is not expected that this underestimation to have influenced trends in BP measurements. Furthermore, the study did not have access to CRA data performed in secondary care. It is possible for another medical specialist, rather than a GP, to be responsible for CVRM in a patient, especially in patients with a history of CVD treated by a cardiologist, or individuals of a younger age with a risk condition. Therefore, it may be that these measurements were performed in secondary care and thus that the proportion of patients with a measurement was underestimated. Lastly, this study was set in a country with an organised healthcare system and mandatory healthcare insurance, which limits the generalisability of the results to countries with a different healthcare structure. The present article does not address the levels of the measured risk factors. Although that may be seen as a limitation, its absence does not affect the findings reported on implementation of the first part of the CVRM guideline; namely, obtaining the information.

### Comparison with existing literature

In the present study, an increase in the proportion of patients with annual BP and cholesterol measurements for patients with DM and/or CVD between 2008–2018 was found. Similar trends have been described in other studies situated in The Netherlands,^16^ New Zealand,^17^ Australia,^18^ and the UK.^19^ A likely explanation for these increasing trends is the growing awareness of primary and secondary prevention as well as the growing target population for CRA with every CVRM guideline update.^10–12,12^ The evidence is expanded by showing that assessment of BP and cholesterol with risk indicated disease groups seems to vary strongly with age. Potentially, such a finding might point towards CVRM control in secondary care in particular, but the finding clearly warrants further validation.

### Implications for practice

The most recent revision of the Dutch CVRM guideline in 2019 recommends CRA in a large population by suggesting the performance of CRA every 5 years in all men aged ≥40 years and post-menopausal women or women aged ≥50 years, but does not clearly state who is responsible for carrying out these recommendations.^10^ The study showed that in routine general practice in the Utrecht area in The Netherlands, 81–97% of men aged ≥40 years and women aged ≥50 years with DM and/or CVD or another indication for regular CRA were measured in 5 years, while patients without an indication were less often measured (56–63%). In The Netherlands, no national organised systematic screening programme is implemented to detect patients at high cardiovascular risk. At present, most practices use an opportunistic screening approach, which depends on the patient visiting the GP practice, on the GP remembering that the patient should receive such an assessment, and on the available time during the consultation. This study shows that usual general practice care already captures a substantial amount of the targeted population. One approach to further improve CVRM in primary care could be to implement a systematic screening programme. A recent study into the effect of a selective cardiometabolic prevention programme showed that an approach involving proactively inviting individuals to come for CVRM screening on top of the opportunistic screening at the GP office was not cost-effective.^20^ The participation rate in this study was 41%^21^ and the most reported reasons for non-response were ‘forgot/no time’ or feeling no need for a test.^22^ Selective non-response is a major problem in the implementation and effectiveness of screening programmes in general. The NHS health check, a CRA programme in the UK, has participation between 32.7–47.0%, although a participation rate of 75% was anticipated.^23–25,25^

Rather than inviting people to participate in a screening program, the opportunistic screening approach can be made more efficient. In The Netherlands, 75% of people visits the GP at least once a year, and this percentage is higher for older patients.^26,27^ If these patients were to be measured during these visits irrespective of the reason for their visit, reaching the goal of measuring all men aged ≥40 years and women aged ≥50 years once every 5 years would be more attainable. Such a change should be proposed in close collaboration with GPs, and must meet preconditions such as a fair compensation and a manageable workload. To support GPs in identifying patients in whom risk factor measurements are indicated, electronic decision support tools have been shown to improve risk factor measurement.^28^ Furthermore, the use of benchmarks can facilitate dialogue between practices, allowing them to learn from each other’s approaches, improve certain indicators for health, and diminish variation between practices, as has been shown by a regional initiative in The Netherlands.^29^

In conclusion, the fairly high frequency of CVRM measurements available in the EHR of patients in primary care suggests an adequate implementation of the CVRM guideline. As nearly all individuals in The Netherlands visit the GP once within a 5-year time window, improvement of CVRM remains very possible, especially in those without a CRA indication.
